# Development of the Eyes

**Published:** 1882-02

**Authors:** 


### DEVELOPMENT OF THE EYES.

1. In children of a few weeks of age, the curvature of the corneais much stronger, the radius therefore much shorter than in the normal eye of adults.

2. Its greatest change takes place, in every case, during the first half of the first year of life.

3. From this period on, the corneal radius gradually increases up to the seventh year.

4. From the seventh year to the twelfth the cornea seems to experience no change in its curvature.

5. In the twelfth and fourteenth years a decided growth in the corneal radius is observed.

6. Between the fifteenth and twentieth years it reaches the same size as is found in adults under normal conditions.—Reuss, *Græfe's Arch.*, 1881. W. W. S.

